# Endoscopic thyroid lobectomy vs Conventional open thyroid lobectomy

**DOI:** 10.12669/pjms.36.4.1604

**Published:** 2020

**Authors:** Mariam Imran, Zahid Mehmood, Muhammad Naseem Baloch, Sehrish Altaf

**Affiliations:** 1 Dr. Mariam Imran, Department of Surgery Ward 25, Jinnah Postgraduate Medical Center, Karachi, Pakistan; 2 Dr. Zahid Mehmood, FCPS FRCS FACS. Department of Surgery Ward 25, Jinnah Postgraduate Medical Center, Karachi, Pakistan; 3 Dr. Muhammad Naseem Baloch, FCPS FACS. Department of Surgery Ward 25, Jinnah Postgraduate Medical Center, Karachi, Pakistan; 4 Dr. Sehrish Altaf, Department of Surgery Ward 25, Jinnah Postgraduate Medical Center, Karachi, Pakistan

**Keywords:** Solitary thyroid nodule, Conventional lobectomy, Endoscopic lobectomy

## Abstract

**Background and Objective:**

Surgical managements for these suspicious nontoxic swellings requires open conventional method of thyroidectomy by neck incisions that can result in prominent scars and immediate risk usually hemorrhage. However new technological innovations came into practiced that include video assisted minimal invasive endoscopy by axillo-breast approach that gives very promising results with excellent cosmesis. In this study, we compared conventional open surgery with minimal invasive endoscopic techniques and associate various complaints and complications that were encountered in surgery.

**Methods:**

Sixty patients were enrolled in this comparative study. It was conducted from period February 2018 to February 2019. The patients were randomized alternatively in two groups. Group-I patients underwent conventional lobectomy while Group-II patients were operated endoscopically, Patients having nodules less than 3cm and Thy 1 and 2 were included in this study. Patient having nodules greater than 3cm, Multinodular goiter, recurrent nodule and Thy 3-6 were excluded from the study.

**Results:**

Patients who underwent endoscopic lobectomy were much more satisfied about scar marks whereas some developed post-operative complications. It included hoarseness of voice in Three (13.62%) patients, two patients developed seroma (9.08%), three patients (13.62%) erythema, whereas no postoperative complications were seen in patients who underwent open thyroid lobectomy. No signs of hypocalcemia noted in both approaches.

**Conclusions:**

The complications with endoscopic approaches are higher but they are minor and resolved spontaneously within maximum period of six weeks. However scar mark satisfaction was much higher in endoscopic lobectomy group.

## INTRODUCTION

Thyroidectomy that is surgical removal of all or part of the thyroid gland may be accomplished for several clinical indications that include malignancy, benign nodules or cysts, suspicious findings on fine needle aspiration biopsy, dysphagia from cervical esophageal compression, or dyspnea from airway compression. Other indications for thyroidectomy include multinodular goiter, Hashimoto’s and other types of thyroiditis, and thyromegaly with significant cosmetic compromise. Additional surgery may involve neck dissection or completion thyroidectomy, based on the extent of disease and final pathology results. Surgeons performing thyroidectomy include otolaryngologists and general surgeons.

Thyroid surgery rates have tripled over the past three decades.^1^ With further advancement minimally invasive thyroid surgeries have evolved over the past decade and several techniques have now been exploited to avoid neck scarring. Among these are axilla-breast endoscopic approaches, gasless axillary approach, trans oral robotic approaches postauricular facelift approach, and transoral vestibular approach.^2^ However the Technological innovation of video-assisted minimally invasive surgery in this era had outstretched the successful attempt by endoscopic thyroidectomy. These are the most preferred as they are related to insignificant pain, minimal tissue trauma, minor wound related complications, shortens hospital stay and draws a great deal of responsiveness because of its cosmetic excellence.^3^

However excision of the thyroid by open conventional method through a midline neck skin incision in the anterior aspect of neck provides good exposure of the anatomy and simplifies safe dissection of underlying vasculature.^4^ While this is standard technique and with experience and expertise of surgeon it results in quick operating time, low morbidity and minimal mortality^5^, while these patients still experienced scar in the neck and several complications from hemorrhages, Intraoperative bleeding that is collectively common in thyroid surgeries because of major vessel in btw restricted visual field.^6^ In circumstances like these, bleeding in operative field prompt various difficulties; postoperatively various complications experienced due to bleeding in particular, that can block the airways include hematoma seroma formation. Therefore, dry surgical field should be provided by performing blood vessel dissection and securing hemostasis.^4^

While excision of thyroid via three ports offers minimum incision i.e 10 mm for camera at anterior axillary line just lateral to nipple and two 5mm port for instruments at medial part of chest^7^ and at anterior axillary line at deltopectoral groove seems more promising as it is cosmetically acceptable and provides good illumination and magnified view. However, risk of complications are analogous to those in open conventional method but somehow more reliant on surgeon proficiency in field of laparoscopy.^7^

The aim of this study was to compare the clinical outcomes between open thyroidectomy vs endoscopic thyroidectomies.

## METHODS

The randomized alternating study was conducted in Jinnah postgraduate medical center, Surgical ward 25. The prerequisite were written and informed consent after explaining the procedure to selected patients.

Sixty (60) patient who underwent open and endoscopic thyroid lobectomy out of which 30 patients went through open approach and 30 went through endoscopic approach from period of February 2018 to February 2019 (NO.F.2-81/2019-GENL/33538/JPMC, Dated: 3-9-2019). All lobectomies were performed by senior consultant Surgeon having 10 years surgical experience of doing thyroid surgeries. Over the period of 12 months, all those patients who were referred to our tertiary care hospital in out-patients departments. There were no limitations to the study we randomly selected 30 patients for minimal invasive endoscopic lobectomy of thyroid gland and remaining 30 patients for open conventional thyroid lobectomy, who fulfilled inclusion criteria.

### Inclusion criteria

1. Patient having nodule size less than <3cm.
2. Abscense of biochemical and ultrasound findings of thyroiditis.
3. Thy 1 and Thy 2 on fine needle aspiration cytology (FNAC).

### Exclusion Criteria

1. Benign nodule of size more than >3cm.
2. Multinogular goiter.
3. Recurrent nodule.
4. Thy3 Thy4 Thy5 Thy6 on fine needle aspiration cytology (FNAC).

### Statistical Analysis

SPSS 16.0 software version used for data analysis. The results were analyzed using fixed- or random-effects models, depending on the heterogeneity involved. Mean and standard deviation were calculated for quantitative variables. Frequencies and percentages were calculated for qualitative variables. Descriptive statistics were used to present the data. Data was stratified to compare different complications among different surgeries.

### Preoperative preparation

Preoperative preparations for both the approaches (open and endoscopic) were same that include vitally stabilizing and hemodynamically optimizing the patient. For that we send biochemical baselines that include complete blood picture, serum urea creatinine electrolytes, serum calcium and thyroid profile making sure patients were in euthyroid state prior to surgery. Consultation from senior anesthetist for fitness apropos general anesthesia was requisite. Preoperative DL (Direct laryngoscope) for vocal cord evaluation and prophylactic antibiotics (once, just before starting operation) were administered.

### Operative procedure of endoscopic thyroid lobectomy

In general anesthesia, surgery was performed. Supine position was made. Sand bag was placed below neck and scapula’s arm of surgery side to reassure the neck in slightly extended position. Three ports were located in such position that first camera port measuring 10mm entered via anterior axillary line just lateral to nipple and two 5mm port for instruments placed at medial part of chest and at anterior axillary line at deltopectoral groove. Above Pectoralis major followed by between Pectoralis major and Platysma a space is fashioned to reach the neck. CO_2_ insufflated with a pressure of 10 mmHg in chest and when entered into neck pressure was reduced to 6mmHG keeping major vessels in mind. Once sternocleidomastoid muscle is identified in neck, lateral retraction was done and strap muscles were identified that were divided vertically to reach the thyroid nodule. Retraction of the strap muscles further laterally to deliver the thyroid nodule. Start dissection of lobe from lower pole to upwards by securing parathyroid gland and recurrent laryngeal nerve. Separate the lobe from trachea and disconnected from opposite lobe. Hemostasis secured. Lobe is taken out by placing into surgical glove. A redivec drain is then placed. However, on a constructive site there were no technical difficulties and limitations found.

### Operative procedure for open thyroid lobectomy

All thyroid lobectomies by open conventional method were performed in general anesthesia. The patient was lying on the operating table in supine position, head end slight elevated to 15 degrees to reduced venous congestion and sandbag placed below neck and scapula region ensuring that the neck is extended increasing the space between the clavicles and the jaws. After all aseptic measures, collar incision was made, subcutaneous tissue and platysma muscle divided. Superior and inferior platysma flaps lifted. Deep cervical fascia opened by vertical incision. Plane created below the fascia to mobilized thyroid gland. Enlarged thyroid nodule identified, Recurrent Laryngeal nerve identified and secured, superior and inferior parathyroid gland identified and secured. Thyroid nodule was mobilized and dissected. Thyroid arteries adjacent to that lobe were ligated. Hemostasis secured, redivac drain was placed and wound was closed in layers. Aseptic dressing applied.

## RESULTS

Sixty patients had lobectomy by open and endoscopic method out of which 30 patients underwent open conventional method whereas the rest 30 patients underwent endoscopic lobectomy of thyroid. The average age of patients was 31.5 years (range 18-45 years). Time duration for both methods depends on the expertise of surgeon however on an average it took two hours for both surgeries. Mostly patients were discharged the next day however the average hospital stay was two days for both method of studies.

One patient underwent endoscopic lobectomy but due to profuse bleeding converted into conventional thyroidectomy by neck incision as it was not controlled by endoscopic technique. Patients who underwent endoscopic lobectomy experienced some post-operative complications that conclude hoarseness of voice that was noted among Three (13.62%) but improved within six weeks. Two (9.08%) Patient developed seroma which was managed by aspiration. Three (13.62%) patients developed neck erthyema that resolved spontaneously within two weeks. No signs of hypocalcemia was noted in this study. Samples for biopsy were sent for all patients, of which six patients (27.24%) diagnosed with papillary carcinoma and far along underwent completion thyroidectomy. A 1-10 scale was made to assess the satisfaction of patient regarding his scar, scar satisfaction was higher in endoscopic group.

**Table-I:** ________________________________________________________

Complications	Endoscopic Thyroid lobectomy	Open Thyroid Lobectomy
Hoarseness of voice	03(13.62%)	None
Seroma	02(9.08%)	None
Neck erythema	03(13.62%)	None
Hypocalcemia	None	None
Cosmesis (scar satisfaction)	6-8	1-2

Patients were orally allowed food after six hours, mobilization was ensured. Redivac drain was removed on first post-operative day and All patients were discharged the next day of surgery. Follow ups were made in out-patients department with biopsy report.

## DISCUSSIONS

Thyroid disorders are most common diseases presented in surgical clinics in our part of world. Nodular thyroid diseases usually treated by surgery. The main concern for delay in treatment is due to visible neck scar, particularly in young girls. They usually inquire regarding alternate treatment options which have no surgery and surgery with no visible scar. To deal with this scenario, surgeons look alternate methods in contrast to conventional method and therefore in 1996 developed endoscopic method of neck surgeries. Various endoscopic thyroid surgery techniques practiced around the world includes; Chest approach, Axillary approach, Axilla breast approach and recently Trans Oral Vestibular Endoscpoic Approach (TOVETA).

Transient recurrent nerve injury was found in three (13.62%) patients who underwent endoscopic approach that tends to be slightly higher than study conducted by Ritesh^8^ (7.96%). this complication can be resolved with the improved visualization of endoscope. The recurrent laryngeal nerve was exposed to reveal its position and course distinctly and conveniently by skilled surgeons, but the thermal damage of ultrasonic scalpel maybe injured the RLN. Owaki et al. suggested that there was no damage to the nerve histologically when the ultrasonic coagulating and cutting systems were used for less than 20 seconds at a distance of 3mm to RLN.^9^ We summarized that functional knife head should be kept away from the nerve and burning time should be reduced as soon as possible while operating near the RLN.^10^ The hoarseness in voice was spontaneously reduced within six weeks whereas the rest 30 patients who underwent lobectomy through open conventional method did not experience hoarseness in voice or any transient damage to recurrent nerve. The revolution in the surgical management of thyroid disorder came after Gagner et al. who conducted his study on patients having hyper parathyroid disorder and undergoing endoscopic approach for subtotal parathyroidectomy for patient with hyperparathyroidism in 1996^11^ Following this Huscher et al., first described complete right thyroid lobectomy in 1997,^12^ these approaches make remarkable changes and dealt with lesser complications and were considered as most preferred approaches.

Minor complications including seroma was noted in two patients(9.08%) but was easily aspirated erythema noted in three patients (13.62%) but was easily manageable and spontaneously reduced in those who underwent endoscopic lobectomy nevertheless no such complications were noted in patients who underwent open conventional procedure. When compared by the study conducted by Chen C where hematoma or bleeding (P=0.15) and seroma (P=0.54) no significant differences were noted between endoscopic and open approach.^13^ Ji young you conducted a study among 205 patients in which one patient developed wound seroma which was treated after several rounds of needle aspiration and no further complications of infections developed.^14^

For patients satisfaction regarding cosmesis a scale was made ranging from one to ten, scoring 10 being the highest regarding patient satisfaction about scar marks on surgical incision site. The results obtained inclines to be inconsistent with those patients who underwent lobectomy of thyroid by open conventional method as they seemed to be much less pleased by surgical neck scar and scored between 1-2 whereas the patients who underwent lobectomies through endoscopic approach seems more satisfied and scored between 6-8. The cosmesis results were excellent in this study and patient satisfaction was much higher, the result was supported by Chung YS et al. in which cosmetic results were excellent in patients who underwent endoscopic thyroid surgeries, no obvious scar marks in neck and patients satisfaction was noteworthy.^15^

In our study hypocalcemia was not noted in any individual but still, hypoparathyroidism is the most feared complication of thyroid surgery. It is most common in total thyroidectomy. In a study conducted in 2002 the reported incidence varies between 0.4% and 13.8% for permanent hypoparathyroidism and is directly correlated with the extent of thyroidectomy. Another study showed that after performing thyroidectomy for large multinodular goiter, temporary hypocalcaemia requiring calcium replacement occurred in 20% of patients. This usually occurs about 36 hours postoperatively. Only up to 3% of patients had persistent hypocalcemia. Chronic hypoparathyroidism with unrecovered normal function after six months was reported in 1.4% of cases and found in more extensive type of surgeryy^16^

## CONCLUSION

After comprehensive comparative analysis between two basic approaches to excised thyroid lobes, the complications with endoscopic approaches are higher but they were minor and resolved spontaneously within maximum period of six weeks. However scar mark satisfaction was much higher in endoscopic lobectomy group.

### Authors’ Contribution

**ZM:** conceived, did statistical analysis & editing of manuscript.

**MI** designed, did data collection and manuscript writing.

**MNB, SA:** did review and final approval of manuscript

**MI, ZM:** Are responsible and accountable for the accuracy or integrity of the work.
